# The Association of MicroRNA-21 with Carotid Artery Disease and Ischemic Stroke: From Pathophysiology to Clinical Implications and Potential Therapy

**DOI:** 10.3390/medsci13030172

**Published:** 2025-09-03

**Authors:** Aleksandar Sič, Marko Atanasković, Alyan Ahmed, Ivan Petrović, Filip Simović, Boris Burnjaković, Una Tonković, Aarish Manzar, Simra Shadab, Selena Gajić, Danka Bjelić, Vidna Karadžić Ristanović, Marko Baralić

**Affiliations:** 1 Faculty of Medicine, University of Belgrade, 11000 Belgrade, Serbiaunaunattt@gmail.com (U.T.); 2 Izola General Hospital, 6310 Izola, Slovenia; atanaskovic.marko5@gmail.com; 3 Faculty of Medicine, University of Novi Sad, 21000 Novi Sad, Serbia; ivan.petrovic@mf.uns.ac.rs (I.P.); boris.burnjakovic@gmail.com (B.B.); 4 Department of Internal Medicine, Ziauddin Medical College, Karachi 75500, Pakistan; alyan.13341@zu.edu.pk (A.A.);; 5 Department of Biomedical Imaging and Image-Guided Therapy, Medical University of Vienna, 1090 Vienna, Austria; 6 Department of Internal Medicine, Liaquat National Hospital and Medical College, Karachi 05444, Pakistan; 7 Nephrology Clinic, University Clinical Centre of Serbia, Pasterova 2 Str., 11000 Belgrade, Serbiavidnakaradzic@gmail.com (V.K.R.)

**Keywords:** biomarkers, therapeutic target, vascular inflammation, endothelial dysfunction, atherosclerosis, asymptomatic carotid disease, cerebrovascular disease, ischemic stroke

## Abstract

Ischemic stroke is one of the leading causes of morbidity and mortality worldwide, with carotid atherosclerosis being its key etiological factor. MicroRNA-21 (miR-21) regulates intracellular signal pathways responsible for vascular changes and ischemic brain injury, and is recognized as a potential diagnostic and prognostic biomarker. It modifies the activity of macrophages (MΦ) and vascular smooth muscle cells, causing inflammation and affecting the stability of atherosclerotic plaques. A deficiency of miR-21 in macrophages stimulates the inflammatory response and plaque growth. It promotes both the synthesis of extracellular matrix, stabilizing the plaque, and the degradation of the fibrin cap, which leads to plaque instability. The effect of miR-21 on endothelial cells differs: it stimulates both NO· synthesis and inflammation. During ischemic stroke, miR-21 demonstrates neuroprotective effects by modulating post-ischemic inflammation and protecting the integrity of the blood–brain barrier. Therapy targeting miR-21 shows potential in experimental models, but it requires cell-specific delivery and precise timing. Further research efforts should focus on the effects of miR-21 on different cell types, as well as the development of new technologies for diagnostic and therapeutic applications.

## 1. Introduction

Ischemic stroke (IS) and carotid atherosclerosis represent major global health challenges, contributing substantially to morbidity, mortality, and long-term disability. According to the Global Burden of Disease (GBD) 2021 study, ischemic stroke affected approximately 69.9 million people worldwide in 2021, with 7.8 million new cases, 3.6 million deaths, and 70.4 million disability-adjusted life years (DALYs) recorded in the same year [1,2]. While age-standardized DALY rates have modestly declined since 1990, reaching around 837 per 100,000 population, the absolute burden continues to rise due to demographic aging and population growth [2].

Carotid atherosclerosis, a key underlying cause of ischemic stroke, is implicated in approximately 15–20% of all cases [3]. Global prevalence estimates suggest that 13–31% of adults exhibit carotid plaques, while moderate-to-severe stenosis is present in about 2–4% [3,4]. Furthermore, studies indicate that 25–27% of adults show increased carotid intima-media thickness and 13–14% have sonographically detectable plaques, even in the absence of clinical symptoms (asymptomatic carotid disease (ACD)) [5,6,7].

Between 1990 and 2021, the number of individuals living with ischemic stroke nearly doubled, rising from ~33 million to ~68 million, respectively [8]. Projections estimate that the annual incidence of ischemic stroke could reach 4.9 million cases by 2030 [9], further intensifying the global burden. Given this growing burden, there is an urgent need for novel biomarkers and therapeutic targets to improve early diagnosis, risk stratification, and treatment outcomes in both carotid artery disease and IS, especially in the group of patients with ACD.

MicroRNA-21 (miR-21), a small non-coding RNA, has emerged as a promising regulator in both vascular pathology and ischemic brain injury. It modulates endothelial function, smooth muscle cell proliferation, matrix remodeling, immune responses, and neuronal survival [10,11,12].

In this review, we explore the dual potential of miR-21 as both a diagnostic and prognostic biomarker, as well as a therapeutic target, in the context of ACD and IS. We aim to provide an overview of its molecular mechanisms across key vascular and neural cell types, highlight its role in disease progression and resolution, and discuss emerging translational approaches that may harness miR-21 for clinical application.

## 2. Materials and Methods

This narrative review was designed to synthesize and critically evaluate the current body of literature on the role of miR-21 in carotid artery disease and ischemic stroke. Unlike systematic reviews, the narrative approach was chosen to accommodate the diverse nature of evidence, including experimental, clinical, and translational studies, and to apply an integrative lens combining translational research and disease pathophysiology frameworks. This allowed for conceptual and thematic exploration.

Although this is a narrative review, we are providing eligibility criteria, as well as a literature search strategy, for clarity, transparency, and a higher level of evidence.

### 2.1. Eligibility Criteria

#### 2.1.1. Inclusion Criteria

- Experimental (animal or clinical) studies evaluating miR-21 as a biomarker or therapeutic target in ischemic stroke and ACD;
- Observational studies linking miR-21 expression to ischemic stroke or carotid disease prognosis;
- Reviews and meta-analyses (only for backward citation searching);
- Case reports demonstrating clinical relevance (e.g., diagnostic or prognostic value).

#### 2.1.2. Exclusion Criteria

- Mechanistic-only studies without diagnostic, prognostic, or therapeutic context;
- Case reports unrelated to diagnosis, treatment, or prognosis;
- Editorials, commentaries, letters to the editor, and non-peer-reviewed abstracts;
- Reviews and meta-analyses.

### 2.2. Literature Search Strategy

A selective literature search was conducted across the following databases: PubMed and Scopus. Key search terms included the following: “microRNA-21”, “carotid artery disease”, “ischemic stroke,” “biomarker,” and “therapeutic target.” Boolean operators were applied to refine search specificity. The search was limited to studies published in English within the last 15 years. In addition, a backward citation search was performed to identify further relevant studies from the reference lists of screened reviews and meta-analyses.

**Figure 1:**
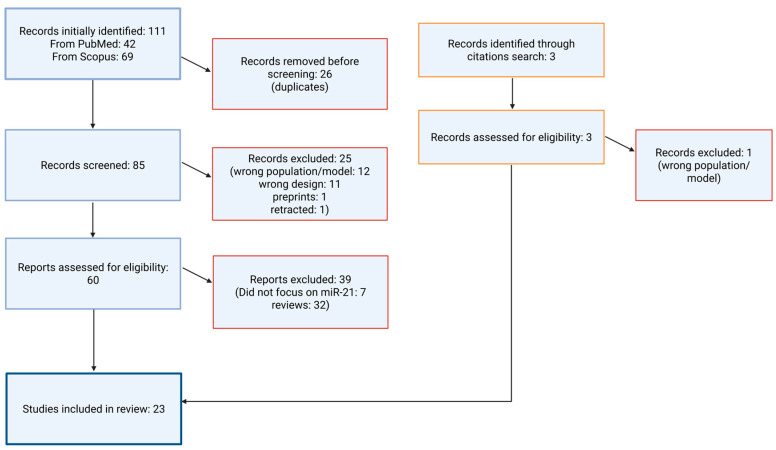
Flowchart illustrating the literature selection process.

A total of 111 citations were initially identified from the electronic databases (PubMed and Scopus). After removing 26 duplicate records, 85 unique citations remained for the title and abstract screening. Two reviewers independently assessed each title and abstract against predefined inclusion and exclusion criteria. Disagreements were resolved by consensus or, if necessary, by adjudication by a third reviewer. Full-text screening was then performed on the citations that met the eligibility criteria after the title and abstract review (Figure 1).

### 2.3. Search Syntax

(a) PubMed

(microRNA-21 or miR-21 or miRNA-21) and (asymptomatic carotid disease (ACD) or ischemic stroke (IS)) and (diagnosis or prognosis or treatment or biomarker or early detection or predictive value or treatment outcome or drug response or targeted therapy).

(b) Scopus

(Title-Abs-Key (“microRNA-21” or “miR-21” or “miRNA-21”) and (Title-Abs-Key (“carotid artery disease” or “ischemic stroke”)) and (Title-Abs-Key (“diagnosis” or “prognosis” or “treatment” or “biomarker” or “early detection” or “predictive value” or “treatment outcome” or “drug response” or “targeted therapy”)) and (Limit-To Language, “English”) (Table 1).

### 2.4. Data Extraction and Thematic Synthesis

A structured data extraction matrix was developed to capture key information from eligible studies, including author/year, study design, population/sample type, pathophysiological mechanism, main findings, and implications related to miR-21 expression. Extracted data were then categorized thematically using two guiding frameworks: the Translational Research Framework (T0–T4) and the Pathophysiological Framework to enable structured synthesis and interpretation across levels of evidence and biological relevance.

### 2.5. Conceptual Framework Application

This narrative review applied a translational (T0–T4) framework and a pathophysiological lens to synthesize current findings on miR-21 in the context of ACD and IS. By mapping miR-21′s role across the translational continuum—from molecular mechanisms (T0) and preclinical experimentation (T1) to early clinical studies (T2) and potential applications in personalized medicine (T3/T4)—we aimed to contextualize its promise and limitations.

The translational framework enabled identification of the maturity stages of miR-21 research, highlighting areas of robust evidence (e.g., preclinical diagnostics) and gaps (e.g., implementation and population-level integration).

In parallel, the pathophysiological framework facilitated synthesis of mechanistic insights, including endothelial dysfunction, vascular remodeling, inflammation, (MΦ), apoptosis, and neurodegeneration. MiR-21′s role in modulating nitric oxide pathways, vascular smooth muscle proliferation, and inflammatory signaling (e.g., STAT3, NF-κβ), as well as apoptosis (e.g., PTEN, PDCD4), was critically examined.

Together, these frameworks supported a comprehensive interpretation of miR-21′s diagnostic, prognostic, and therapeutic potential, while also identifying research gaps to inform future translational directions.

### 2.6. Review of the Literature

The studies included in the literature review are listed in Table 2.

## 3. Mechanistic Role of miR-21 in Atherosclerosis

Atherosclerosis is a chronic inflammatory condition of the arterial wall involving lipid accumulation, immune cell infiltration, and fibrous plaque development. The microRNA miR-21 is dysregulated in several cell types within atherosclerotic plaques and exerts pleiotropic effects that influence both plaque progression and stability [36].

In the following sections, we discuss the major mechanistic roles of miR-21 in regulating vascular inflammation, vascular smooth muscle cell (VSMC) proliferation, endothelial dysfunction, and plaque vulnerability.

## 4. MiR-21 in Vascular Inflammation

Inflammation is a driving force in atherogenesis and plaque destabilization. MiR-21 is highly expressed in macrophages—in fact, it is one of the most abundant miRNAs in these immune cells and functions as an important feedback regulator of inflammation [36]. Its expression is induced by pro-inflammatory stimuli such as toll-like receptor (TLR) ligands (e.g., LPS) and tumor necrosis factor-α (TNF-α) [37,38]. One of the best-characterized targets of miR-21 in macrophages is programmed cell death 4 (PDCD4), a protein that enhances NF-κβ activation and inhibits the anti-inflammatory cytokine interleukin-10 (IL-10) [38,39]. By suppressing PDCD4, miR-21 limits NF-κβ –mediated transcription of inflammatory cytokines while facilitating IL-10 production. This regulation drives macrophages toward an anti-inflammatory (M2-like) phenotype marked by increased IL-10 and arginase-1 (Arg1) expression [40]. Consistently, overexpression of miR-21 in macrophages attenuates TLR4/NF-κβ driven responses, decreasing IL-6 secretion and enhancing IL-10 release in response to LPS stimulation [41].

In vivo, hematopoietic deficiency of miR-21 disrupts this regulatory balance. Mice lacking miR-21 in bone marrow–derived cells, such as ApoE^−/− or LDLr^−/− models, develop significantly larger and more inflamed atherosclerotic plaques, accompanied by increased macrophage apoptosis [42]. A bone marrow transplant study further confirmed that LDLr^−/− mice reconstituted with miR-21–deficient hematopoietic cells exhibited a greater plaque burden and inflammation compared to controls, highlighting the protective and anti-inflammatory role of miR-21 in atherosclerosis [43].

Similarly, Canfrán-Duque et al. (2017) demonstrated that miR-21 knockout in macrophages accelerates atherogenesis and promotes vascular inflammation and necrotic core formation, whereas miR-21 sufficiency helps limit plaque necrosis [37]. MiR-21 deficiency led to upregulation of MAP kinase-kinase-3 (MKK3), hyperactivation of p38 MAPK and CHOP (a mediator of endoplasmic reticulum stress and apoptosis), and impaired cholesterol efflux due to post-translational downregulation of the transporter ABCG1 [14,41,44]. These changes increase macrophage susceptibility to apoptosis and reduce lipid clearance, thereby exacerbating plaque expansion and instability.

In summary, miR-21 plays an important role in resolving vascular inflammation by reducing macrophage-driven inflammatory signaling and cell death.

## 5. MiR-21 and Vascular Smooth Muscle Cell Proliferation

During atherogenesis, vascular smooth muscle cells (VSMCs) in the arterial media undergo phenotypic switching from a contractile to a synthetic, proliferative phenotype, contributing to neointimal lesion formation and fibrous cap development. MiR-21 is a well-characterized pro-proliferative microRNA in VSMCs across various cardiovascular diseases, including atherosclerosis. In response to vascular injury or mechanical stress, miR-21 expression is upregulated in VSMCs, promoting their dedifferentiation and abnormal proliferation [45,46].

In a rat model with miR-21 knockdown, Sun et al. demonstrated that miR-21 enhances VSMC proliferation and migration through activation of the PI3K/Akt and ERK signaling pathways, thereby accelerating atherosclerotic lesion development [47]. Conversely, inhibition of miR-21 has been shown to suppress pathological VSMC growth. In a carotid balloon-injury model mimicking angioplasty-induced neointimal hyperplasia, miR-21 expression was significantly increased in the injured arterial wall during neointima formation. Therapeutic knockdown of miR-21 resulted in a marked reduction in neointimal thickening. This effect was associated with the depression of key miR-21 targets involved in limiting cell survival and proliferation, most notably PTEN, a negative regulator of the PI3K/Akt pathway, and Bcl-2, an apoptosis modulator. As a result, VSMC proliferation was attenuated, apoptosis increased, and overall lesion growth was blunted [47]. These findings identify miR-21 as a critical pro-survival and pro-proliferative factor in VSMCs that contributes to intimal hyperplasia.

From a therapeutic standpoint, targeting miR-21 in the vessel wall could reduce excessive VSMC accumulation, a major contributor to luminal stenosis in carotid atherosclerosis. However, the role of VSMCs extends beyond lesion expansion. These cells also contribute to plaque stability by synthesizing extracellular matrix proteins that reinforce the fibrous cap. Thus, miR-21′s effects on plaque biology are context-dependent: while its upregulation promotes VSMC-driven lesion growth, it may simultaneously support the structural integrity of advanced plaques [48]. Complete genetic ablation of miR-21 in vascular wall cells could impair reparative VSMC responses and weaken fibrous cap formation.

Current evidence strongly suggests that miR-21 exerts a net pro-atherogenic effect during early lesion development by driving unchecked VSMC proliferation. Therefore, precise and context-sensitive modulation of miR-21 expression may be necessary to achieve therapeutic benefits without compromising plaque stability.

**Figure 2:**
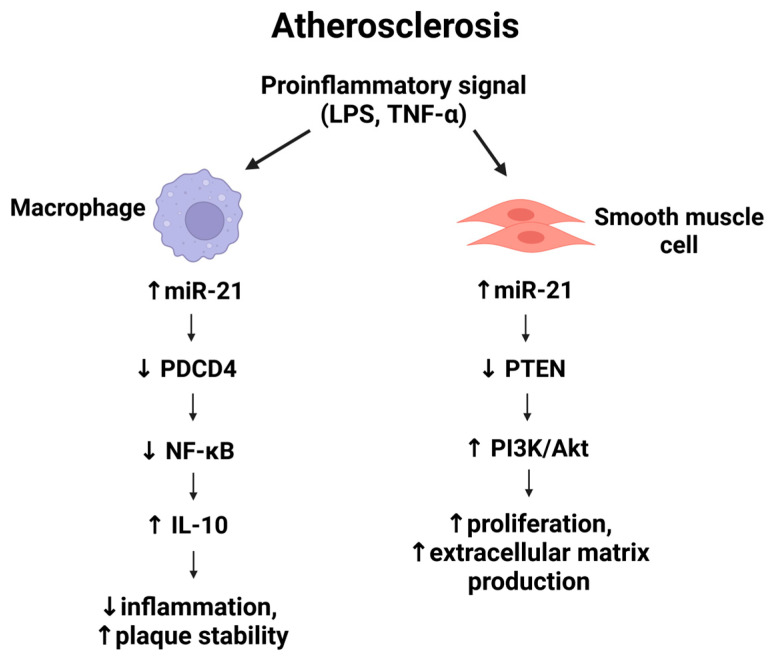
Schematic representation of miR-21–mediated signaling in macrophages (MΦ) and vascular smooth muscle cells during atherogenesis. Created with the BioRender online application, available at https://app.biorender.com/ (accessed 10 July 2025).

Figure 2 illustrates the pro-inflammatory activation of miR-21 in macrophages and vascular smooth muscle cells, highlighting its divergent roles in promoting anti-inflammatory cytokine production and cellular proliferation via PDCD4/NF-κβ and PTEN/PI3K/Akt pathways, respectively.

## 6. MiR-21 and Endothelial Dysfunction

Endothelial cells (ECs), which line the interior of blood vessels, are also direct targets of miR-21 regulation [49]. Endothelial dysfunction is a critical initiator of atherosclerosis [50]. Laminar blood flow in straight parts of arteries provides atheroprotection, whereas disturbed flow at branch points induces endothelial inflammation and plaque formation [50]. Interestingly, miR-21 is involved in the endothelial responses to different shear stress patterns, acting as a mediator of both protective and pathological processes depending on the hemodynamic context. Under oscillatory or low shear stress, which is pro-atherogenic, endothelial miR-21 is upregulated and contributes to a maladaptive inflammatory phenotype [51,52]. MiR-21 induced by oscillatory flow was shown to directly target PPARα, a transcription factor with anti-inflammatory effects in ECs. The loss of PPARα due to high miR-21 levels leads to increased activation of AP-1 (a pro-inflammatory transcription factor) and upregulation of endothelial adhesion molecules and chemokines. Consequently, monocytes (Mo) adhere more readily and migrate into the intima, seeding plaque development. In this scenario, miR-21 forms part of a positive feedback loop: disturbed flow → miR-21 up → PPARα suppression and VCAM-1/MCP-1 induction → monocyte recruitment and inflammation → further miR-21 elevation [53,54,55]. By contrast, under unidirectional high shear stress (as in normal arterial regions), miR-21 appears to play a protective role in maintaining endothelial homeostasis. Laminar flow also increases EC expression of miR-21, but in this setting miR-21′s downstream effects include PTEN inhibition and Akt/eNOS pathway activation, which boosts NO· production [56]. MiR-21 in laminar flow conditions was found to reduce endothelial apoptosis and promote NO-mediated vasoprotection via the PI3K/Akt/eNOS cascade [57,58]. In vitro, overexpression of miR-21 in endothelial cells (HUVECs) led to enhanced eNOS phosphorylation and NO· release and reduced apoptosis [57]. Notably, miR-21 overexpression also slowed EC proliferation and migration via targeting of RhoB, an effect that could limit excessive angiogenesis [59].

Overall, in established atherosclerosis-prone regions (disturbed flow), miR-21 tends to promote endothelial activation, whereas in stable flow regions it aids endothelial resilience [60]. Targeting miR-21 for therapy would need to account for this complexity to avoid impairing the endothelium’s adaptive responses to shear stress.

## 7. MiR-21 and Plaque Instability

Plaque instability is the propensity of an atherosclerotic lesion to rupture or erode and trigger thrombosis, and is influenced by the plaque’s cellular composition and extracellular matrix integrity. Key features of unstable plaques include a large necrotic core, thin fibrous cap, and active inflammation with matrix-degrading enzymes [61]. MiR-21 impacts several of these features. On one hand, as discussed, miR-21 helps limit MΦ apoptosis and promotes efferocytosis (clearance of dead cells) via its anti-apoptotic, pro-resolving actions. By reducing MΦ death and necrotic debris, miR-21 can mitigate necrotic core expansion, which would favor plaque stability. Consistently, miR-21 deficiency in atherosclerotic mice was associated with larger necrotic cores and more inflamed, vulnerable plaques [37]. On the other hand, miR-21 can contribute to cap degradation by boosting matrix metalloproteinase activity in MΦ. Fan et al. (2014) [62] identified miR-21 as part of a “plaque instability signature” in human coronary lesions: MΦ within unstable (thin-cap) plaques had elevated miR-21 expression and produced higher levels of MMP-9, a collagenase that weakens fibrous caps. They found that miR-21 directly targets RECK (reversion-inducing cysteine-rich protein with Kazal motifs), an endogenous inhibitor of MMPs. Thus, when miR-21 is overexpressed in macrophages, RECK is suppressed and MMP-9 secretion increases, leading to aggressive matrix degradation. In vitro, overexpression of miR-21 in human MΦ caused a marked rise in both pro-MMP9 and active MMP9 levels, an effect that was mimicked by RECK knockdown. Clinically, patients with non-calcified, rupture-prone plaques showed significantly higher miR-21 levels in plaque macrophages (MΦ), but paradoxically lower circulating miR-21 levels, potentially due to sequestration in lesions or consumption, alongside elevated plasma MMP-9 [62]. Their findings implicate the miR-21–RECK–MMP-9 axis in fibrous cap degradation and highlight miR-21 as a potential marker of plaque vulnerability. Indeed, miR-21 (together with miR-221) has been proposed as a circulating marker of unstable carotid plaques and future stroke risk [18].

Such context-dependent effects highlight the need for a better understanding. miR-21′s net effect on plaque stability likely depends on the stage of atherosclerosis and the relative contribution of its actions in different cell types.

## 8. MiR-21 in Ischemic Stroke Models

Ischemic stroke, often a downstream consequence of atherosclerotic disease, triggers a cascade of pathophysiological processes in the brain, including excitotoxic cell death, oxidative stress, inflammation, and blood–brain barrier (BBB) disruption [63]. Current data suggest that miR-21 is dynamically regulated after stroke and that it modulates several injury response pathways in the acute and subacute phases of ischemia [64,65]. Clinical observations have shown that circulating miR-21 changes rapidly in stroke patients. In the hyperacute stage of ischemic stroke, plasma miR-21 levels tend to be lower than in controls, and interestingly, this reduction correlates with greater initial neurological deficits (higher NIH Stroke Scale scores). Within the ensuing days after a stroke, however, miR-21 expression rises significantly in the blood and can remain elevated for an extended period [66,67,68]. One study reported that by day 7 after stroke onset, patients had markedly increased serum miR-21, especially those with co-existing carotid atherosclerosis [18]. The early dip and later surge of miR-21 may reflect acute consumption or tissue uptake of miR-21, followed by induction of miR-21 as part of the recovery/inflammatory resolution phase. In the brain tissue itself, miR-21 is induced in specific cell populations after ischemia [69]. Buller et al. (2010) first showed that miR-21 is upregulated in neurons within the peri-infarct (ischemic penumbra) region after experimental stroke [30]. Neuronal miR-21 appears to act as a pro-survival factor: overexpressing miR-21 in cultured neurons subjected to oxygen-glucose deprivation (an in vitro stroke model) significantly inhibited apoptosis, in part by directly suppressing the pro-apoptotic Fas ligand (FASLG). This anti-apoptotic effect translated to smaller infarcts and improved cell survival in vivo, suggesting that miR-21 upregulation in neurons protects against ischemic cell death [30]. Similarly, in a neonatal hypoxic–ischemic brain injury model, Liu et al. (2020) observed that miR-21 expression was downregulated in the injured brain [70], and restoring miR-21 via a mimic injection reduced infarct volume and improved neurobehavioral outcomes. The benefit was attributed to miR-21 targeting and downregulating CCL3 (a pro-inflammatory chemokine), which in turn reduced NF-κβ signaling in the brain [70]. This further supports the notion that miR-21 ensures neuroprotection by both anti-apoptotic and anti-inflammatory mechanisms.

MiR-21 also plays a role in modulating post-stroke inflammation and BBB integrity [71]. Stroke causes a robust inflammatory reaction involving resident microglia and infiltrating immune cells [72]. MiR-21 is known as a regulator of microglial polarization; for instance, it can drive microglia toward an anti-inflammatory state in other CNS injury models [73,74]. In the context of cerebral ischemia, one key target of miR-21 is the interleukin-6 receptor (IL-6R). A recent study found that miR-21-5p directly binds to the 3′UTR of IL6R mRNA, inhibiting its expression and thereby reducing IL-6–mediated inflammatory signaling. Notably, stroke patients exhibited an inverse relationship between miR-21-5p and IL-6R in their peripheral blood, with miR-21-5p levels being significantly decreased. In contrast, IL-6R levels were elevated, suggesting that the loss of miR-21′s regulation may exacerbate inflammation in acute stroke. By introducing miR-21-5p (or blocking IL-6R) in cellular models of stroke, researchers could alleviate inflammation-induced damage [13]. In addition to inflammation, BBB disruption is a major factor in stroke pathology, leading to edema and secondary injury [75]. MiR-21 appears to influence BBB stability via effects on endothelial and supporting cells in the neurovascular unit. In a rat ischemia-reperfusion (I/R) model, Yao et al. (2018) showed that miR-21 upregulation has protective effects on the BBB. Specifically, elevated miR-21 in the post-stroke brain inhibited the p38/MAPK signaling pathway by targeting MAP2K3 (MKK3), which led to reduced production of inflammatory cytokines, attenuated BBB permeability increase, and less vasogenic edema [76]. Treated rats showed improved neurological scores, linking the molecular effect to functional outcome. This finding dovetails with the earlier atherosclerosis studies where miR-21 suppressed MKK3-p38 signaling in MΦ [37], suggesting a common pathway by which miR-21 reduces inflammation-induced tissue damage in both vascular walls and brain tissue [76]. Paradoxically, there is also evidence that miR-21 can contribute to BBB damage under certain conditions: Deng et al. (2013) observed that cerebral ischemia caused a significant upregulation of miR-21 along with MMP-9 in the rat brain, and administration of an antagomir-21 after stroke decreased MMP-9 levels and reduced BBB leakage and lesion size [77]. They proposed a calcium-(Ca^2+^)-dependent pathway in which ischemic signals trigger ERK activation via miR-21, thereby increasing MMP-9 expression. In this scenario, miR-21 was driving MMP-9–mediated matrix degradation (similar to its role in plaque instability), and blocking miR-21 proved beneficial [77]. These seemingly conflicting observations can be explained by the temporal dynamics and cell-type specificity of miR-21 activity: during the acute phase of stroke, early upregulation of miR-21 in neurons or reactive astrocytes contributes to MMP-9–mediated injury. In contrast, its sustained elevation in the subacute and chronic phases primarily facilitates recovery by attenuating inflammation and promoting cell survival [78,79]. Nevertheless, the exact window during which miR-21 augmentation versus inhibition is optimal remains to be determined.

Finally, miR-21 is a versatile regulator in ischemic stroke. It can protect neurons from apoptotic death, modulate the inflammatory milieu, and preserve vascular integrity; however, under certain conditions, it may also exacerbate deleterious protease activity. This makes miR-21 an intriguing but complex therapeutic target in stroke.

**Figure 3:**
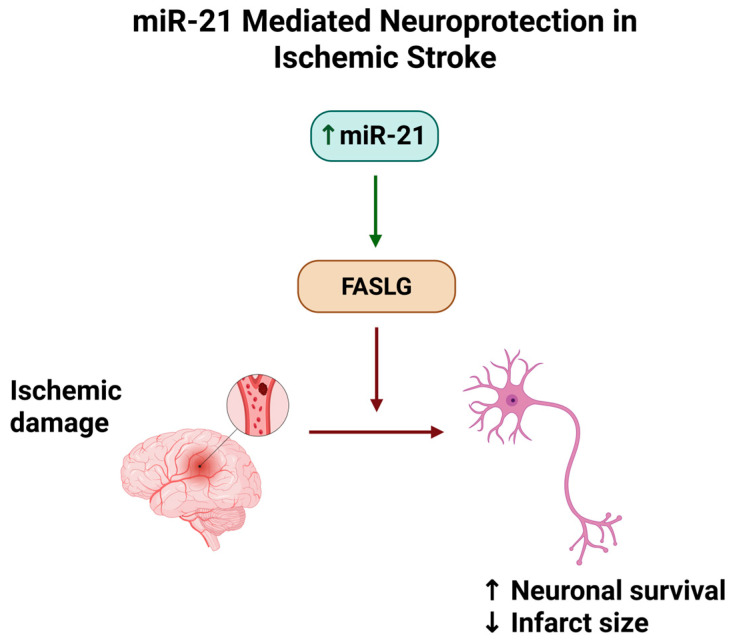
MiR-21–mediated neuroprotection in ischemic stroke. Upregulation of miR-21 after ischemic damage suppresses FASLG expression, reducing neuronal apoptosis and infarct volume. Created with the BioRender online application, available at https://app.biorender.com/ (accessed 10 July 2025).

Below, Figure 3 shows the neuroprotective mechanism of miR-21 in ischemic stroke, focusing on the suppression of FASLG and the consequent reduction in neuronal apoptosis following ischemic injury.

## 9. Therapeutic Modulation of miR-21

Given its involvement in multiple pathogenic processes, miR-21 has emerged as an interesting potential therapeutic target in cardiovascular and cerebrovascular diseases. Strategies to modulate miR-21 generally fall into two categories: miR-21 inhibition (using antisense oligonucleotides or “antagomirs” to knock down miR-21 activity) and miR-21 augmentation (using miR-21 mimics or gene therapy to increase its level in target cells). Both approaches have shown promise in preclinical studies, though their utility depends on disease context.

For example, an LNA-based miR-21 inhibitor was able to slow myocardial fibrosis and subsequent hypertrophy and heart failure in a mouse model. A similar model has been used in clinical trials to slow renal fibrosis in glomerulopathies and various stages of chronic kidney disease (CKD) to prevent the development of end-stage kidney disease (ESKD) and the need for kidney replacement therapy (KRT), demonstrating that therapeutic silencing of miR-21 is feasible in humans [80]. Due to the diverse, cell-specific functions of miR-21 in atherosclerosis, the decision between its inhibition and augmentation remains complex. On one hand, inhibiting miR-21 in VSMCs and MΦ could reduce plaque growth and instability by curbing SMC proliferation and MMP-9 secretion [43,62]. Local delivery of miR-21 inhibitors effectively reduces neointimal hyperplasia in injured arteries and may represent a viable strategy for limiting carotid plaque expansion [27]. On the other hand, augmenting miR-21 in lesional MΦ might enhance resolution of inflammation and promote plaque stabilization by increasing efferocytosis and IL-10 production [40]. The bone-marrow transplant experiments indicate that global miR-21 inhibition in all MΦ would likely aggravate plaque inflammation, an unwanted effect [81,82]. Therefore, any therapeutic intervention targeting miR-21 in ACD would likely need to be cell-type-specific or timed appropriately.

In IS, the preclinical data largely support miR-21 enhancement as a neuroprotective strategy [68,83]. Several studies have demonstrated that treatments that elevate miR-21 in the brain, either via viral vectors, pharmacological upregulation, or exosomal delivery, lead to better outcomes after experimental stroke [30,70]. Another promising avenue is using stem cell–derived extracellular vesicles (EVs) loaded with miR-21 [84]. Exosomes, which are nanosized vesicles naturally enriched in miRNAs, can cross biological barriers like the BBB and deliver their cargo to recipient cells with high efficiency [85]. Because exosomes are inherently biocompatible and non-immunogenic, they are being explored as vehicles for miRNA therapy in stroke and other CNS disorders [86]. In support of this concept, studies have shown that mesenchymal stem cell–derived exosomes with high miR-21 content confer neuroprotection and enhance angiogenesis in ischemic models [87]. Modulating miR-21 in donor stem cells can alter the therapeutic potency of their exosomes. For instance, knocking down miR-21 in exosome-producing cells abolishes some of the exosomes’ beneficial effects on recipient tissues [87]. Such insights implicate a potential strategy of exosome-based miR-21 delivery to the post-stroke brain.

## 10. Biomarker Potential of miR-21

Many clinical studies (≥T2) included in this review have investigated the role of miR-21 as a biomarker for the diagnosis and prognosis of ACD or IS [13,15,20,22,23,26,35]. For example, Zhan et al. found that hsa-miR-21-5p was significantly decreased in the peripheral blood of hospitalized IS patients compared to healthy controls (*p* < 0.001), suggesting its potential as a diagnostic biomarker [13]. In contrast, Xiang et al. reported that serum miR-21 was increased in IS patients, although the miR-21 gene rs1292037 T > C polymorphism was not significantly associated with IS risk [15].

Furthermore, Zhang et al. investigated serum miR-21-5p levels in 123 patients with acute focal neurological signs within 24 h of onset, including 84 AIS patients, 39 TIA patients, and 30 healthy controls [20]. They found that miR-21-5p was significantly higher in AIS patients compared to TIA patients and controls (*p* < 0.001), and it was further elevated in patients with poor prognosis (mRS 3–6) (*p* < 0.05). MiR-21-5p levels also correlated positively with NIHSS and mRS scores, with AUC values of 0.710 for distinguishing AIS from TIA and 0.641 for predicting poor 30-day outcomes, suggesting its prognostic potential.

Vibo et al. found that serum miR-21 levels were significantly higher at stroke onset in patients with cryptogenic stroke but not in those with large artery atherosclerosis [22]. MiR-21 expression during the acute phase was markedly higher than at the 1-year follow-up (*p* < 0.0001), and higher miR-21 levels were significantly associated with greater stroke severity. However, the study was limited by a small sample size and unequal group distribution, and emphasized the need for further research to clarify miR-21′s role in cryptogenic stroke.

Wu et al. showed that serum miR-21-5p was significantly upregulated in IS patients and could help distinguish IS from TIA. ROC analyses indicated its potential as a predictive and discriminative biomarker for IS and TIA, as well as an indicator of post-TIA neurological deficit severity and stroke risk.

Zhou et al. reported that plasma miR-21 levels were significantly lower in ACI patients than in controls and, in contrast to Zhang et al., found a strong negative correlation between plasma miR-21 levels and NIHSS scores within the first day, suggesting miR-21 may serve as a potential early marker for ACI [26].

In contrast to both of these studies, Bahar et al. found that serum miR-21 expression was significantly higher in AIS patients than in controls (*p* < 0.001). Still, they showed no correlation with proinflammatory cytokines, stroke severity (NIHSS), or outcome (mRS) [35]. They concluded that larger, multicenter studies are needed to clarify its diagnostic value.

Variations in timing, sample type, and patient characteristics are likely responsible for the contrasting results observed across studies.

The timing of sample collection for the assessment of miRNA expression is likely to significantly influence the measured levels and their role in ischemic stroke. Circulating miR-21 can fluctuate dynamically during the acute phase due to evolving tissue injury, inflammation, and reperfusion processes [88]. As mentioned above, Zhou et al. observed plasma miR-21 within the first day after stroke and reported decreased levels [26], whereas Zhang et al. assessed serum miR-21-5p within 24 h of symptom onset in acute patients and observed increased levels [20]. Differences in sample collection timing may partly explain these contrasting findings. Edwardson et al. reported that it is more meaningful to collect samples near the time of inpatient rehabilitation rather than during acute hospitalization, aiming to capture molecular changes associated with spontaneous biological recovery rather than initial injury [89].

In addition to the timing of sample collection, the type of sample itself (serum vs. plasma) can have a major effect on miR-21 expression. For example, the type of sample in the study by Zhang et al., where higher levels of miR-21-5p were detected, was serum [20], while in the study by Zhou et al., which found decreased levels of miR-21, it was plasma [26]. There are discrepancies in studies regarding how sample type affects levels of different platelet- or erythrocyte-derived miRNAs in blood [90]. The primary hypothesis that blood coagulation could increase levels of certain platelet-derived miRNAs during serum preparation is opposed by the contrasting results of Wakabayashi et al. [91] and the proposed theory that during preparation of serum, exosomes with platelet-derived miR-NAs were absorbed, and/or during preparation of plasma, those were released into blood [90]. However, this and other studies did not, to the best of our knowledge, evaluate the influence of sample type on specific miR-21 in blood. Therefore, it may still not be possible to recommend a single sample type for the evaluation of miR-21. These two sample types should be compared carefully between studies, taking into account preparation methods and timing of preparation after blood collection.

Lastly, patient demographics may also play a role in miR-21 trends in blood. According to the study by Accardi et al., miR-21-5p levels were found to increase with age, although the rate of increase slowed over time [92]. The highest levels were observed around ages 60–65, after which they declined toward levels similar to those seen in younger individuals. No statistically significant associations were detected with sex, BMI, or smoking status in this study. However, other studies have reported that sex-related differences in miRNA regulation may exist and may partly arise from variations in the miRNA processing machinery [93]. Estrogen (E2), for example, can modulate miRNA maturation at a post-transcriptional level by attenuating the conversion of pri-miRNAs to pre-miRNAs through its interaction with the Drosha complex. In line with this, miR-21 has been shown to be suppressed by ERβ in the normal female mouse heart, whereas this effect is absent in males. In addition to age and gender, differences in comorbidities and stroke severity should also be considered. Therefore, careful adjustment in statistical analyses and matching of study groups with controls should be a priority.

In summary, although many studies suggest the potential of miR-21 as a biomarker for diagnosis, differential diagnosis, and prognosis, further clinical research is needed. Future studies should include larger sample sizes, standardized control groups, and consistent sample types, while also accounting for the timing of sampling, as miRNA expression may vary depending on disease stage and context.

## 11. Discussion and Limitations

Despite encouraging results, there are important challenges and considerations in translating miR-21–targeted therapies for clinical use.

First, off-target effects need careful evaluation. MiR-21 has hundreds of mRNA targets and is expressed in many cell types, so systemic manipulation could have unintended consequences (perturbing tumor suppressor pathways, since miR-21 is an oncomiR in cancer). Achieving cell-specific delivery (using targeted nanoparticles or cell-specific promoters in gene therapy) will be key to minimizing side effects [94,95,96,97,98,99].

Second, the timing of the intervention appears critical. As discussed above, miR-21 can play opposite roles at different stages of disease or injury. For example, inhibiting miR-21 very early after myocardial infarction attenuates adverse remodeling; however, inhibiting it later may impair scar formation and healing [43]. A similar principle may apply to stroke: a narrow window might exist where augmenting miR-21 yields maximal benefit, whereas outside that window it might be neutral or even harmful (by promoting MMP activity too late) [100,101]. Therefore, understanding the temporal profile of miR-21 in each condition is vital to inform therapeutic timing.

Third, delivery efficiency remains a hurdle. While antagomirs and mimics work well in rodents (often delivered by direct injection or high doses), human translation will require improved delivery systems to ensure the oligonucleotides reach the intended tissue in adequate concentrations. Novel delivery systems, including lipid nanoparticles, conjugated chemical carriers, or cell-derived exosomes, are under active investigation to address this. Encouragingly, ongoing development of miRNA therapeutics in other diseases provides a roadmap; for instance, several miRNA drugs (against different targets) have entered clinical trials, indicating that safety and pharmacokinetic issues, while non-trivial, can be overcome [102,103,104].

It is also worth noting the biomarker aspect of miR-21 in translation. Even if direct targeting of miR-21 proves complex, measuring miR-21 in patient samples could have clinical utility. The fact that serum or plasma miR-21 levels are associated with plaque instability and stroke severity suggests that miR-21 could serve as a risk stratification tool. For example, a high circulating miR-21 (and miR-221) signature might identify patients with carotid plaques that are more likely to cause stroke [18,105]. As miR-21 is also abundant in platelets and other blood cells [106], careful interpretation is required; however, standardized assays for miR-21 may soon complement imaging and clinical scores in guiding carotid intervention or stroke management. In the context of asymptomatic carotid disease, the central clinical dilemma remains how to identify individuals at the highest risk of future stroke who may benefit from revascularization. Current data do not support the use of miR-21 as a stand-alone biomarker for this purpose. However, circulating miR-21, particularly as part of multi-miRNA panels and when integrated with imaging markers of plaque instability, may enhance risk stratification beyond traditional clinical criteria [27,107,108].

The absence of standardized reference ranges in healthy individuals presents another challenge in translating miR-21 into clinical practice. Reported baseline miR-21 levels vary widely between studies due to differences in sample type (serum, plasma, whole blood, extracellular vesicles, tissue), RNA extraction methods, normalization strategies (choice of endogenous controls, for example), and detection platforms (qRT-PCR, microarray, next-generation sequencing). Also, age, sex, ethnicity, diet, physical activity, circadian rhythm, and comorbid conditions can all influence miR-21 expression, even in apparently healthy individuals [109,110]. Recent evidence shows that miR-21 expression increases with aging across multiple tissues, including vascular endothelium, skeletal muscle, and immune cells. In older adults, elevated miR-21-5p levels have been reported in circulating extracellular vesicles and senescent endothelial cells, where it contributes to endothelial dysfunction. In skeletal muscle, age-related upregulation of miR-21 impairs satellite cell differentiation and regeneration, particularly under pro-inflammatory conditions characterized by elevated IL-6 and TNF-α. In the immune system, increased miR-21 in naïve CD4^+^ T cells skews differentiation toward short-lived effector phenotypes, reducing memory formation and contributing to immunosenescence. These findings highlight that aging is not only a confounder in interpreting baseline miR-21 levels but may also represent a biological context in which miR-21 actively drives vascular, muscular, and immune decline, potentially amplifying atherosclerotic and cerebrovascular risk [111,112,113].

The absence of large, multicenter studies with harmonized methodologies prevents researchers from establishing universally accepted cut-off values for “normal” expression. Without such baseline standardization, comparing results across studies or defining thresholds for disease detection remains challenging [109,110].

## 12. Conclusions

MiR-21 plays an important and context-dependent role in atherosclerosis, asymptomatic carotid disease, and ischemic stroke. Its regulation of key molecular pathways, such as PDCD4, PTEN, MAP2K3, RECK, FASLG, and IL-6R, affects vascular inflammation, extracellular matrix remodeling, endothelial function, and neuronal survival. In atherosclerosis, miR-21 contributes to both plaque progression and stabilization, while in stroke, it can show either protective or harmful effects depending on timing and cell type. Although preclinical studies suggest that modulating miR-21 may offer therapeutic benefits, challenges remain regarding precise delivery, timing, and potential off-target consequences. Beyond therapy, circulating miR-21 shows promise as a biomarker for plaque instability and stroke prognosis. Refining our understanding of cell-specific effects and leveraging advanced technologies to guide the clinical translation of miR-21-based diagnostics and treatments should be the priority of future research efforts in this domain.

## Figures and Tables

**Table 1:** Search strategies used for PubMed and Scopus databases.

Database	Search Syntax
PubMed	(microRNA-21 OR miR-21 or miRNA-21) and (carotid artery disease or ischemic stroke) and (diagnosis or prognosis or treatment or biomarker or early detection or predictive value or treatment outcome or drug response or targeted therapy)
Scopus	Title-Abs-Key (“microRNA-21” or “miR-21” or “miRNA-21”) and Title-Abs-Key (“carotid artery disease” or “ischemic stroke”) and Title-Abs-Key (“diagnosis” or “prognosis” or “treatment” or “biomarker” or “early detection” or “predictive value” or “treatment outcome”or “drug response” or “targeted therapy”) and Limit-To Language, “English”)

**Table 2:** Summary of included studies on microRNA-21 in ischemic stroke and carotid artery disease (*n* = 23). AIS—acute ischemic stroke; ACI—acute cerebral infarction; BBB—blood–brain barrier; BCCAO—bilateral common carotid artery occlusion; BMSC—bone marrow–derived mesenchymal stem cell; BMSC-Exos—exosomes derived from BMSCs; CAD—coronary artery disease; CA—carotid atherosclerosis; CIRI—cerebral ischemia–reperfusion injury; Cryp—cryptogenic stroke; Du—Governing Vessel (traditional Chinese medicine meridian); EC—endothelial cell; EVs—extracellular vesicles; FASLG—Fas ligand; HMEC-1—human microvascular endothelial cells; HUVECs—human umbilical vein endothelial cells; ICAM-1—intercellular adhesion molecule 1; IL—interleukin; IMT—intima-media thickness; IV—intravenous; LAA—large-artery atherosclerosis; lncRNA—long non-coding RNA; MAT2B—methionine adenosyl-transferase II beta; MCAO—middle cerebral artery occlusion; MEG3—maternally expressed gene 3; miR/miRNA—microRNA; mRS—modified Rankin Scale; MWM—Morris water maze; NBAT1, TUG1—long non-coding RNAs; NF-κβ—nuclear factor kappa β; NIHSS—National Institutes of Health Stroke Scale; OGD—oxygen–glucose deprivation; PBMC—peripheral blood mononuclear cells; PDCD4—programmed cell death protein 4; REST—RE1-silencing transcription factor; ROC—receiver operating characteristic; ROS—reactive oxygen species; SD rats—Sprague-Dawley rats; SMC—smooth muscle cell; TIA—transient ischemic attack; TLR4—toll-like receptor 4; TNF-α—tumor necrosis factor alpha; UDCA—ursodeoxycholic acid; VEGF, VEGFR2, Ang-1, Tie-2—pro-angiogenic factors; VSMC—vascular smooth muscle cell.

Author/Year	Study Type	Population/ Model	Translational Stage (T0–T4)	Disease Focus	Pathophysiological Mechanism	Main Findings	Implications
Zhan et al., 2023 [13]	Observational clinical + in vitro functional study	Ischemic stroke patients (*n* = 60), healthy controls (*n* = 23); OGD-treated HMEC-1 cells	T2	Ischemic stroke	miR-21-5p suppresses IL-6R; downregulated in patients; IL-6R linked to ischemic injury.	miR-21-5p ↓ in patients (*p* < 0.001); overexpression improves cell viability and reduces apoptosis.	Potential biomarker and therapeutic target for reperfusion injury.
Mohammed et al., 2022 [14]	Clinical observational human and in vitro study	A total of 60 AIS patients vs. 60 healthy controls; HMEC-1 cells under OGD ± miR-21-5p mimic/inhibitor	T1	Acute ischemic stroke	Four ncRNAs linked to AIS: TUG1 ↑, NBAT1 ↑, miR-21 ↑, miR-335 ↓; correlations with lipid and thyroid profile).	NBAT1: 100% sens/spec; TUG1: 80% sens; miR-335: 73.3% sens, 100% spec.	Novel non-invasive biomarkers; link to atherosclerosis and thyroid function.
Xiang et al., 2017 [15]	Genetic association case–control	A total of 592 ischemic stroke patients vs. 456 healthy controls	T2	Ischemic stroke	miR-21 rs1292037T > C polymorphism; circulating miR-21; miR-126G > A linked to ↓ stroke risk.	miR-21 ↑ in stroke (*p* < 0.001); miR-126G > A polymorphism associated with reduced stroke risk.	miR-126/miR-21 expression may serve as biomarkers and therapeutic targets.
Liu et al., 2021 [16]	Clinical observational correlation study	A total of 170 AIS patients vs. 100 high-risk controls; PBMCs, cytokine ELISA; 36-month follow-up for recurrence	T1	Acute ischemic stroke	lnc-MEG3 ↑ and miR-21 ↓ regulate inflammation and vascular microenvironment.	lnc-MEG3 ↑ (AUC 0.874), miR-21 ↓ (AUC 0.889) in AIS; correlation with recurrence risk.	Diagnostic/prognostic value; potential targets for reducing post-stroke inflammation and recurrence.
Li et al., 2019 [17]	Preclinical animal + in vitro study	Male SD rats (*n* = 126), MCAO model; SH-SY5Y and HMEC-1 cells under OGD/reoxygenation ± miR-21-3p	T0	Ischemic stroke	ADMSCs suppress miR-21-3p → MAT2B ↑ → apoptosis and inflammation ↓; BBB improved.	ADMSCs reduced infarct size, apoptosis, IL-1β/IL-6/TNF-α; miR-21-3p inhibition increased viability.	Targeting miR-21-3p/MAT2B via ADMSCs or inhibitors may improve stroke recovery.
Tsai et al., 2013 [18]	Observational human study	A total of 167 ischemic stroke patients; 66 carotid atherosclerosis subjects; 157 healthy controls	T1	Ischemic stroke, atherosclerosis	Circulating miR-21 increases with stroke severity.	miR-21 significantly elevated in stroke and atherosclerosis vs. controls; independent predictor.	miR-21 may serve as a biomarker for stroke and carotid atherosclerosis.
Lei et al., 2024 [19]	In vivo animal preclinical intervention	Male SD rats, MCAO model; 4 groups (Sham, Surgery, Acupuncture, Nimodipine); 15-day acupuncture; cognitive testing	T0	Ischemic stroke	Acupuncture ↓ REST → ↑ miR-21-3p → ↓ PDCD4 → ↓ apoptosis, ↑ cognitive function.	Acupuncture reduced REST, increased miR-21-3p, decreased cytokines/apoptosis, and improved cognition.	Acupuncture may aid post-stroke recovery via REST/miR-21-3p axis; potential complementary therapy.
Zhang et al., 2023 [20]	Clinical observational biomarker study	A total of 84 AIS patients, 39 TIA patients, 30 healthy controls	T3	Acute ischemic stroke, TIA	Serum miR-21-5p upregulated with severity and poor outcome.	miR-21-5p ↑ in AIS vs. TIA and controls; correlated with NIHSS and mRS; AUC = 0.710 for AIS vs. TIA.	miR-21-5p may help differentiate AIS from TIA and predict short-term outcome.
Wang, 2018 [21]	Clinical biomarker observational study	A total of 143 IS patients (by phase) + 24 controls; plasma exosomes analyzed	T1	Ischemic stroke	Exosomal miR-21-5p/miR-30a-5p dynamically change with stroke stage.	miR-21-5p ↑ in subacute and recovery phases; best AUC ~0.7 for later phases.	Exosomal miRNAs may aid diagnosis, staging, and timing of therapy in stroke.
Vibo et al., 2024 [22]	Clinical observational study	A total of 73 young patients with cryptogenic and LAA stroke; blood samples at onset and 1-year follow-up	T2	Ischemic stroke (Cryp vs. LAA)	Inflammatory gene/miRNA upregulation (e.g., miR-21) in cryptogenic stroke.	Cryp stroke: ↑ miR-21, ICAM1, TNF, IL1B during acute phase vs. follow-up; correlated with hs-CRP and severity.	Suggests role of miR-21 in inflammation and severity of cryptogenic stroke.
Wu et al., 2017 [23]	Clinical cohort + biomarker study	Serum: 50 IS patients, 50 controls (screening); 177 IS, 81 TIA, 42 controls (validation)	T1–T2	Ischemic stroke, TIA	Circulating miRNAs reflect neurovascular injury, inflammation, stress response.	miR-21-5p ↑ in IS vs. TIA; logistic regression and ROC: predictive and discriminative potential.	miR-21-5p may help assess stroke severity and risk after TIA.
Korvenlaita et al., 2023 [24]	Preclinical animal study (biomarker focus)	Male Balb/c mice, permanent MCAO model	T0–T1	Ischemic stroke, hypoxia	Hypoxia rapidly ↑ miR-21a-5p in EVs; majority remains in non-EV form.	miR-21a-5p = most deregulated neuronal miRNA; EV and non-EV miR-21 correlate with worse outcome.	Circulating miR-21a-5p may predict disability and guide early post-stroke care.
Hu et al., 2022 [25]	Preclinical in vivo + in vitro study	Stroke mouse model; HUVECs for angiogenesis assays	T0–T1	Ischemic stroke recovery	BMSC-derived exosomes deliver miR-21-5p → ↑ angiogenesis via VEGF, Ang-1, Tie-2.	BMSC-Exos ↓ infarct size, ↑ neurological function and micro vessel density; miR-21-5p enhanced EC migration.	BMSC-Exos may enable cell-free stroke therapy by promoting angiogenesis through miR-21-5p.
Zhou et al., 2014. [26]	Mechanistic + biomarker study	In vitro OGD (N2A cells); plasma from 68 ACI patients and 21 controls	T1–T2	Acute cerebral infarction	miR-21 (anti-apoptotic) and miR-24 (pro-apoptotic) regulate Bcl-2, XIAP.	Plasma miR-21 and miR-24 were lower in ACI; both negatively correlated with NIHSS.	miR-21 and miR-24 may serve as early diagnostic/prognostic biomarkers and therapeutic targets.
Jin et al., 2018 [27]	Experimental (mechanistic + therapeutic)	Human plaques (*n* = 20); Apoe−/−, miR-21−/−, and Apoe−/−miR-21−/− mice; local miR-21 delivery	T0–T1	Atherosclerosis (carotid)	miR-21 modulates SMC proliferation, macrophage activity; REST–miR-21–REST feedback loop.	miR-21 ↓ in unstable plaques; miR-21 deficiency → plaque rupture; local miR-21 stabilized plaques.	miR-21 is a potential therapeutic target for plaque stabilization in atherosclerosis.
Raskurazhev et al., 2020 [28]	Observational human case–control study	A total of 25 carotid atherosclerosis patients vs. 11 controls; leukocyte miRNA from blood plasma	T1	Carotid atherosclerosis	miR-21 inhibits Pdcd4 in VSMCs and macrophages, reducing apoptosis and inflammation.	miR-21-5p/3p downregulated in CA; expression correlated with anti-atherogenic mechanisms.	miR-21 may have diagnostic and protective roles in CA; microRNAs could be future therapeutic targets.
Lopez et al., 2022 [29]	Experimental animal intervention study	Adult and aged male/female C57BL/6 mice; transient MCAO model	T1	Ischemic stroke	miR-21 mimic suppresses pro-apoptotic, inflammatory, and autophagy mRNAs.	miR-21 mimic ↓ infarct volume and improved motor recovery; effective via intracerebral or IV delivery.	Supports miR-21-based neuroprotection as a promising post-stroke therapeutic strategy.
Buller et al., 2010 [30]	Experimental in vivo + in vitro	Male Wistar rats with embolic MCAO; cultured cortical neurons	T1	Ischemic stroke	miR-21 upregulation inhibits FASLG, reducing neuronal apoptosis.	miR-21 protected neurons post-stroke by directly targeting pro-apoptotic FASLG.	miR-21/FASLG axis represents a potential therapeutic target for stroke treatment.
Moradi et al., 2021 [31]	Preclinical therapeutic intervention	Rat model of cerebral ischemia–reperfusion injury (BCCAO)	T1	Ischemic stroke	Wild blueberry extract modulates miR-21 and miR-146a; reduces iNOS, TNF-α, and oxidative stress.	Extract ↑ miR-21/miR-146a, ↓ inflammation and oxidative damage; preserved hippocampal neurons.	Diet-derived compounds like blueberry extract may activate protective miRNA pathways post-stroke.
Tu et al., 2020 [32]	Preclinical therapeutic study	SH-SY5Y neuronal cells + mouse model of ischemic stroke	T1	Ischemic stroke	Pterostilbene ↑ miR-21-5p → ↓ PDCD4 → ↓ apoptosis and infarct size.	Pterostilbene reduced infarct size and neuronal apoptosis; effect mediated by miR-21-5p upregulation.	Suggests therapeutic potential of Pterostilbene via miR-21-5p modulation in stroke treatment.
Unal et al., 2025 [33]	Observational gene expression	A total of 50 patients with CAD and carotid atherosclerosis; samples from plaques and internal mammary arteries	T1	Carotid and coronary atherosclerosis	Dysregulated miRNAs drive plaque inflammation and remodeling.	miR-21-5p ↑ 22-fold in plaques vs. healthy tissue (*p* = 0.0001.)	miR-21-5p may serve as biomarker and therapeutic target in atherosclerosis.
Huang et al., 2020 [34]	Preclinical in vivo + in vitro study	SD rats (carotid ligation model); cultured rat vascular smooth muscle cells (VSMCs)	T1	Atherosclerosis, restenosis	UDCA ↓ miR-21 → ↑ PTEN → ↓ AKT/mTOR → ↓ VSMC proliferation and migration.	UDCA suppressed intimal hyperplasia and VSMC growth; miR-21 overexpression reversed UDCA effects.	UDCA may prevent vascular remodeling via the miR-21/PTEN/AKT/mTOR pathway.
Bahar et al., 2024 [35]	Case–control study	A total of 64 acute ischemic stroke patients and 22 age-matched controls (Indonesia)	T2	Acute ischemic stroke	miR-21 and cytokines (TNF-α, IL-10, ICAM-1, CCL5) involved in inflammation.	miR-21 significantly ↑ in stroke; cytokines elevated; no correlation with NIHSS or mRS scores.	miR-21 may be diagnostic marker, but not predictive of short-term stroke severity/outcome.

## Data Availability

Data are contained within the article.
